# Diet-Independent Positive Effects of a Multi-Species Probiotic on the Growth Performance and Resistance against *Vibrio parahaemolyticus* in White Leg Shrimp

**DOI:** 10.3390/ani13030331

**Published:** 2023-01-17

**Authors:** Christina Gruber, Dan Bui-Chau-Truc, Jutta C. Kesselring, Ngoc Diem Nguyen, Benedict Standen, Silvia Wein

**Affiliations:** 1 DSM Animal Nutrition & Health, BIOMIN Holding GmbH, Erber Campus 1, 3131 Getzersdorf, Austria; 2 DSM Animal Nutrition & Health, BIOMIN Vietnam Co. Ltd., Aquaculture Center for Applied Nutrition, Street 11-Nong Lam University Campus, Quarter 6, Linh Trung Ward, Ho Chi Minh City 720371, Vietnam; 3 DSM Animal Nutrition & Health, BIOMIN Vietnam Co. Ltd., Binh Duong Site, No. 6, Street 20, Vietnam Singapore IIA, IZ, Vinh Tan Ward, Tan Uyen 75409, Vietnam

**Keywords:** probiotic, functional feed, white leg shrimp, resistance to diseases, *Vibrio parahaemolyticus*, aquaculture

## Abstract

**Simple Summary:**

The aquaculture industry is facing several challenges, including water quality, stocking density and disease outbreaks due to bacterial pathogens. Pathogens and challenging conditions in aquaculture are common problems that cause mortality, reduce growth performance and consequently lead to high economic losses. To prevent those losses, antibiotics are often used for treatment or prophylaxis. With the increasing concern for antibiotic resistance and the demand to reduce the application of antibiotics, alternative solutions are needed. Incorporating probiotics in the diet can be one of the solutions to support feed efficiency as well as the resilience of the animals to pathogen pressure. The results of two experiments demonstrate that probiotic feed additives are promising strategies to improve shrimp production and provide increased protection against bacterial infection, independent of the diet formulation.

**Abstract:**

Probiotic feed additives can support the gut health of shrimp and thereby improve performance, production efficiency and disease resistance. Two experiments in white leg shrimp aimed to investigate the effects of a multi-species probiotic feed supplement (AquaStar^®^, 3 g/kg feed, Biomin GmbH, Getzersdorf, Austria) in feed formulations with different marine meal levels (32% and 15%) on growth performance and resistance against *Vibrio parahaemolyticus*. Juvenile shrimp were stocked in a recirculating aquaculture tank system at a density of 20 shrimp/46.8 L and were fed diets with and without the probiotic supplementation for 8 weeks. Afterwards, a bath immersion with *V. parahaemolyticus* was performed and mortality was observed over a period of 14 days. Independent of the diet formulation, probiotic supplementation significantly improved the survival rate of the shrimp and the specific growth rate while decreasing feed consumption and feed conversion ratio when compared to the control (*p* ≤ 0.042). After the *Vibrio* immersion challenge, mortality was significantly decreased by 13.33% with probiotic supplementation in the high marine meal diet experiment (*p* = 0.042) and numerically decreased by 11.67% in the low marine meal diet experiment (*p* = 0.133). Overall, the results suggest that the beneficial effects of the probiotic can occur independently of the diet formulation.

## 1. Introduction

One of the main challenges that prevents the further growth of crustacean aquaculture is disease outbreak due to bacterial pathogens, which are generally favored by insufficient water quality and a high stocking density. As a result, the aquaculture industry is still relying on antibiotics for treatment or prophylaxis to prevent economic losses.

For example, Acute Hepatopancreatic Necrosis Disease (AHPND) has caused great economic losses to all shrimp-producing countries in Asia. The causative agents of AHPND are specific strains of *Vibrio parahaemolyticus* that harbor a plasmid (pVA) encoding the Pir-like binary toxin genes PirA^VP^ and PirB^VP^. The binary toxin PirA/B^VP^ causes massive sloughing of tubule epithelial cells and finally results in the death of the infected shrimp [1]. Due to the high concern for antibiotic resistance and the resulting demand to reduce the application of antibiotics, alternative solutions are needed to help overcome the disease-related challenges.

In addition to disease control, the industry is also facing challenges with regards to sustainability. Replacing the finite marine resources with plant ingredients is pivotal in reducing the overall environmental impact of shrimp production [2]. Hence, the solutions for disease control should ideally also help to reduce the environmental impact of shrimp production to enable the growth of the sector.

Probiotics have been gaining acceptance as disease-controlling agents in aquaculture in the last few years [3] and can also support feed efficiency and growth performance of the animals [4]. The effectiveness of probiotics depends on the timing, dosage, administration, species and strain [5]. The dietary supplementation of mixed probiotic products containing *Lactobacillus reuteri*, *Pediococcus acidilactici*, *Enterococcus faecium* and *Bacillus subtilis* have previously been reported to improve the feed efficiency of shrimp, compete with *V. parahaemolyticus* in an intensive shrimp culture system [6] and increase the immune readiness of shrimp [7]. In vitro, *E. faecium*, *L. reuteri* and *P. acidilactici* were shown to inhibit pathogen adhesion to primary epithelial cell culture [8]. The *B. subtilis* strain of the same mixed probiotic product was demonstrated to degrade the binary PirA/B^VP^ toxin in vitro and to improve the survival of *Artemia* after immersion with the purified AHPND toxin [9]. Incorporating probiotics in reduced marine meal diets might support feed efficiency as well as resilience to pathogen pressure.

The aim of this study was to assess the effects of a commercially available multi-species probiotic, AquaStar^®^ (a mix of live *Bacillus subtilis*, *Enterococcus faecium*, *Lactobacillus reuteri* and *Pediococcus acidilactici*) on the growth performance and resistance against *Vibrio parahaemolyticus*, independent of the feed formulations. For this reason, we chose two feed formulations: a good-quality diet with a high marine meal content (i.e., 32%, of which 24% is fish meal) and a less costly diet with a low marine meal content (i.e., 15%, of which 9% is fish meal).

## 2. Materials and Methods

Two separate experiments testing either a 32% or a 15% marine meal diet (Table 1) were performed at the Biomin Aquaculture Center for Applied Nutrition (Ho Chi Minh City, Vietnam). Specific pathogen resistant (SPR) post-larvae (PL9-10) *Litopenaeus vannamei* from a local hatchery were held for a 40-day acclimation period under recirculating water conditions (31.0–32.5 °C, 15–18 ppt salinity, pH 7.5–8, dissolved oxygen (DO) 5–6 ppm, total ammonia nitrogen (TAN) 0.25 ppm, nitrites < 0.5 ppm, alkalinity > 150 ppm). For the measurements, we used a handheld oxygen meter (Oxi 3210 Set 1 DO Meter WTW 2BA201, WTW, Weilheim, Germany), a handheld pH meter with pH sensor InLab Expert Pro (S20-K SevenEasy™ pH, Mettler Toledo, Columbus, OH, USA) and API^®^ test kits (API Inc., Chalfont, PA, USA).

Both experiments took place in tanks with dimensions 60 cm × 30 cm × 47 cm, 26 cm water in a recirculating aquaculture system (RAS), testing the recommended supplementation level of 3 g/kg AquaStar^®^ (*Bacillus subtilis*, *Pediococcus acidilactici*, *Enterococcus faecium*, *Lactobacillus reuteri* at a total cell count of 1 × 10^9^ cfu/g, Biomin, Austria) applied post-pelleting. In a previous study testing AquaStar^®^ [7], we evaluated the amount of viable probiotic bacteria in shrimp feed 2 weeks after feed preparation and confirmed that they maintain their viability in the expected amount of shrimp feed.

For the first experiment, 400 shrimp (mean body weight ± standard deviation: 2.27 ± 0.01 g) were randomly placed in 20 tanks (20 shrimp/tank). A total of 2 experimental groups (Table 1) were randomly assigned to the tanks: 12 replicates without probiotic supplementation (CON) and 8 replicates with probiotic supplementation (Pro-3g) of a high marine meal diet. For the second experiment using a low marine meal diet, 240 shrimp (mean body weight ± standard deviation: 1.78 ± 0.01 g) were also randomly placed in 12 tanks (20 shrimp/tank) and assigned to the 2 experimental groups (Table 1) with 6 replicates each. Compared to the high marine meal diet, the cost of the low marine meal diet was approximately 35% lower.

During the 56-day feeding trials, shrimp were fed to near satiety 6 times per day. Feeding behavior, feed intake and mortality were recorded for each tank daily to estimate the amount of feed provided in the subsequent meals. The shrimp were weighed 56 days after feeding the experimental diets and the feed consumption during the trial period was quantified. Feed conversion ratio (FCR) was calculated as the proportion of the total feed consumption to the shrimp weight gain per tank. Specific growth rate (SGR) was calculated according to the formula:

$$SGR=\frac{ln\left(final\;mean\;shrimp\;weight\right)-\ln\left(initial\;mean\;shrimp\;weight\right)}{time\;\left(days\right)}\times 100$$

After the 56-day feeding trial, a subsequent challenge experiment with *Vibrio parahaemolyticus* was conducted. A total of 10 shrimp in the intermolt stage were randomly selected from each tank and transferred to reciprocal 40 L plastic tanks in a dedicated challenge room. Each plastic tank was equipped with a small biofilter. Shrimp were acclimatized to the new conditions for 3 days in both experiments, to ensure that no mortality occurred after the transfer. Before applying the bacterial challenge, shrimp were challenged with 24 h immersion in a 20 ppm ammonia solution to increase the susceptibility to the infection, followed by 1 h immersion 1 × 10^6^ cfu/L with *Vibrio parahaemolyticus* (AHPND positive strain, purchased from Fish Pathology Lab, Faculty of Fisheries, Nong Lam University, Ho Chi Minh City, Vietnam). After 1 h of bacterial immersion, 50% of the water from each challenge tank was exchanged with fresh salt water.

In the experiment with the high marine meal diet, 3 replicates of the control diet group received only the ammonia challenge and the remaining 4 replicates of the control and the 6 replicates of the probiotic group were submitted to the full challenge procedure. Due to the higher mortality in the experiment with the low marine meal diet, 2 replicates of the control group and 3 replicates of the probiotic group were submitted to the full challenge procedure.

Mortality was monitored and recorded for 14 days. Shrimp were observed at least twice daily. Moribund or dead shrimp were removed and examined for gross external clinical signs of disease. During the challenge period, shrimp were fed twice daily with the same feed allocated to each group during the performance trial (Table 1), at a rate of 2 % of the overall biomass divided between 2 meals.

To determine the cause of death, 3–4 dead shrimp in good condition were selected per group at day 7 after the bacterial challenge and sent to the Shrimp Vet laboratory (Minh Phu Aquamekong Co. Ltd., Ho Chi Minh City, Vietnam) for AHPND testing via real-time PCR. Similarly, we analyzed 1 sample of 4–5 dead animals 7 days after the challenge in the replicates that received only the ammonia challenge and 1 sample of 4–5 animals across the groups before the *Vibrio* challenge to rule out cross-contamination.

Boxplots were used to visually inspect the data distribution, variability and outliers. The model assumptions (normality, homoscedasticity and independence) were inspected via the residual plots. Independent samples *t*-test (parametric), Kruskal–Wallis test (non-parametric) and Poisson regression (parametric) were used to test for differences between the treatments. Kaplan–Meier survival analysis was used to investigate differences in survival time after challenges among the treatments. All statistics were conducted using the statistical program R (version 3.5, R Foundation for Statistical Computing, Vienna, Austria). The significance level was defined as *p* < 0.05, and *p* values *p* ≥ 0.05 but <0.10 were considered tendencies.

## 3. Results

Independent of diet, the probiotic supplementation significantly increased the survival rate of the shrimp and the specific growth rate while at the same time decreasing feed consumption and feed conversion ratio when compared to the control (*p* ≤ 0.042, Table 2). When the high marine meal diet was supplemented with the probiotic, body weight after 8 weeks of feeding the experimental diets was on average 6% higher and the body weight gain 12% higher than in the control (*p* ≤ 0.032).

**Figure 1:**
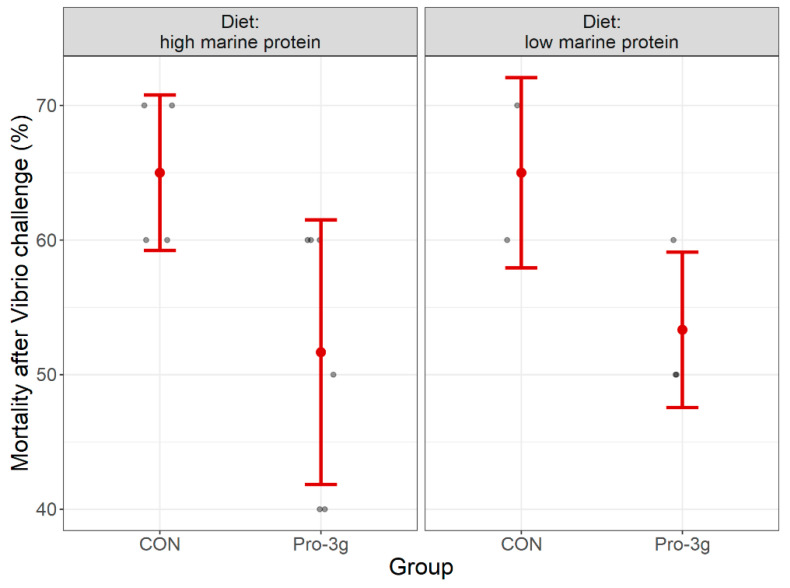
Mortality (%) of white leg shrimp, *Litopenaeus vannamei*, 14 days after the immersion challenge with *Vibrio parahaemolyticus*. The group mean is represented by the red dot and standard deviation is indicated using the red whiskers. The dark dots are the mortality of the specific replicates per group. CON = diet without probiotic supplemented, Pro-3g = diet with probiotic supplementation, i.e., AquaStar^®^ (3 g/kg).

The percentage of dead shrimp 14 days after immersion with *Vibrio parahaemolyticus* was significantly decreased by 13.33% with probiotic supplementation in the high marine meal diet experiment (*t*-test, *p* = 0.042) and numerically decreased by 11.67% in the low marine meal diet experiment (*p* = 0.133, Figure 1).

In the high marine meal experiment, we additionally examined a negative control group for the challenge, i.e., the animals only received the ammonia immersion challenge but not the *V. parahaemolyticus* challenge. The mortality 14 days after the ammonia immersion challenge without *V. parahaemolyticus* immersion was 6.67 ± 5.77% (mean ± standard deviation), which was significantly lower than the mortality in the group with the *V. parahaemolyticus* immersion (65 ± 5.48%, *t*-test, *p* < 0.001).

To determine the cause of death after the *Vibrio* immersion challenge, the animal samples were collected during the challenge trial to test for detection of Acute Hepatopancreatic Necrosis Disease (AHPND). The real-time PCR analysis did not detect AHPND in the pooled samples of shrimp sacrificed prior to the immersion challenge or in the pooled samples of shrimp that died during the experiment in the group that received only the ammonia challenge. However, the 4–5 dead shrimp sampled 3–7 days after the bacterial challenge were AHPND-positive in both experiments, indicating that the cause of mortality was the bacterial infection.

## 4. Discussion

Probiotics can provide a defense against pathogens [3] and improve shrimp growth performance, feed conversion ratio and the general survival of animals [4]. The results of the two experiments supported these claims. Using a repeatable bath immersion challenge model with *V. parahaemolyticus* in the two experiments demonstrated that the groups supplemented with the probiotic feed additive had a more than 10% lower mortality 2 weeks after the bacterial challenge compared to the non-supplemented control group, independent of the marine meal inclusion level of the basal diet.

The findings are likely based on general mechanisms. Predominant mechanisms are likely the nutritional effects of the multi-species probiotic. In both experiments, all shrimp receiving a multi-strain probiotic preparation, independent of the underlying feed formulation, showed improved survival and specific growth rates paralleled by decreased feed consumption and FCR compared to the control-fed animals. This is likely due to the nutritional effects of the probiotic supplementation and specifically the ability to improve protein usage for growth or tissue deposition. Gluten hydrolysis has previously been described for isolates of *E. faecium*, *P. acidilactici* and *B. subtilis* [10]. Even though shrimp do not utilize glucose monomers well, they have a 92% usage efficiency for starch. The alpha amylase activity of the probiotics will therefore not help the shrimp. However, hydrolyzing the gluten molecules could have a positive effect on the shrimp with regards to feed efficiency and growth rate. Generally, bigger shrimp have a better defense against any type of challenge than smaller individuals. Additionally, the particular strain of *B. subtilis* utilized in the probiotic preparation is known to enhance the immune readiness of rainbow trout [8] and can degrade PirA/B^VP^ in vitro as well as in vivo in the brine shrimp, *Artemia franciscana* [9]. The whole probiotic mix has previously been shown to enhance the defense efficiency of shrimp [7]. As described by [8], energy is also more efficiently directed to defense through immune regulation (i.e., dampening of the immune system via IL-10 upregulation) by lactic acid bacteria. Modifying the virulence by competing with the *Vibrio* species to reach a critical mass to switch on virulence (quorum sensing), or by employing quorum quenching mechanisms as described for several *Bacillus* sp., might also play a role in the positive probiotic effect [11,12].

After feeding the experimental diets for 8 weeks (before the start of the challenge), a 76.67% survival rate was observed when the shrimp received the high marine meal control diet, whereas a 50% survival rate was observed when feeding the low marine meal control diet with only 9% fish meal. Shrimp feed manufacturers have decreased the inclusion level of fishmeal almost by half during the last 20 years, to around 11–23% with a further downwards trend [2]. However, replacing fish meal with plant-based protein in shrimp feed often results in increasing mortality if the diet is not additionally supplemented with essential amino acids or other functional additives [10,11,12]. Hence, the low survival rate in the low marine meal control group is likely due to malnutrition and nutrient deficiencies. Interestingly, the surviving shrimp in the low fishmeal diet with only 9% fish meal had a generally good growth rate. Further breeding efforts as well as further advances in feed formulation are needed in order to increase the survival rates of shrimp on low marine meal diets.

## 5. Conclusions

The results of the two experiments demonstrate that probiotic feed additives are promising strategies to improve shrimp production and provide increased protection against *V*. *parahaemolyticus* infection, independent of the marine meal level in the diet.

Incorporating probiotics in reduced marine meal diets might support feed efficiency as well as resilience to pathogen pressure. Further studies are needed to clarify the contribution of each component of the probiotic mix in order to optimize the probiotic product formulation for low marine meal diets as they are being fed now as compared with 10 to 20 years ago. Furthermore, to clarify the mode of action, additional analyses to investigate gut and water microbiota, immune defense and digestibility are needed.

## Figures and Tables

**Table 1:** Feed composition of the test diets.

Experiment	(1) High Marine Meal Diet		(2) Low Marine Meal Diet	
Compound Feed GP Trial	CON	Pro-3g	CON	Pro-3g
Ingredient	Inclusion Rate [%]	Inclusion Rate [%]	Inclusion Rate [%]	Inclusion Rate [%]
Local fish meal 60% protein	24.00	24.00	9.00	9.00
Fish soluble			3.00	3.00
Krill meal	8.00	8.00	2.00	2.00
Squid meal			1.00	1.00
Hemoglobin powder			3.00	3.00
Soybean meal 46% protein	30.50	30.50	35.00	35.00
Corn gluten			8.21	8.21
Wheat gluten			2.50	2.50
Rice bran			9.51	9.51
Fish oil	2.50	2.50	2.00	2.00
Liquid lecithin	1.00	1.00	2.59	2.59
Whole wheat	29.00	29.00	18.43	18.43
Vitamin premix	1.00	1.00	0.02	0.02
Monocalcium Phosphate	2.00	2.00	1.64	1.64
Limestone	0.45	0.45	1.00	1.00
Salt	0.50	0.50	0.30	0.30
Lysine HCL	0.30	0.30	0.00	0.00
Methionine	0.08	0.08	0.24	0.24
Cholesterol	0.08	0.08	0.10	0.10
Choline Chloride 50%	0.33	0.33	0.10	0.10
Threonine	0.20	0.20	0.10	0.10
Betaine			0.10	0.10
Taurine			0.10	0.10
Vitamin C	0.006	0.006	0.06	0.06
AquaStar^®^	0 g/kg	3 g/kg	0 g/kg	3 g/kg
Moisture (EC 152/2009)	3.52 g/100 g		4.21 g/100 g	
Crude fiber (AOCS Ba-6a-05)	3.05 g/100 g		3.83 g/100 g	
Crude fat (TCVN 4331:2001/ ISO 6492:1999)	5.3 g/100 g		6.33 g/100 g	
Protein (APAC 2001.11)	40.78 g/100 g		39.75 g/100 g	
Carbohydrates (calculation)	36.4 g/100 g		37.69 g/100 g	
Ash (EC 152/2009)	14 g/100 g		12 g/100 g	
Gross energy (calculation)	85.18 kJ/100 g		87.64 kJ/100 g	

CON = diet without probiotic supplemented, Pro-3g = diet with probiotic AquaStar^®^ (3 g/kg).

**Table 2:** Growth performance of shrimp after 8 weeks of feeding the experimental diets with or without the supplementation of a multi-species probiotic at 3 g/kg feed. Values are means ± standard deviation.

Experiment	(1) High Marine Meal Diet		(2) Low Marine Meal Diet	
	CON	Pro-3g	CON	Pro-3g
Initial mean shrimp weight per tank (g)	2.26 ± 0.01	2.27 ± 0	1.78 ± 0.01	1.78 ± 0.01
Final mean shrimp weight per tank (g)	9.79 ± 0.61 ^a^	10.40 ± 0.46 ^b^	9.69 ± 0.76	9.77 ± 1.40
Weight gain (g)	6.82 ± 0.56 ^a^	7.65 ± 0.40 ^b^	6.10 ± 0.65	6.75 ± 1.31
Feed intake (g shrimp^−1^)	11.66 ± 0.92 ^a^	10.98 ± 0.30 ^b^	13.71 ± 1.25 ^a^	11.71 ± 1.40 ^b^
FCR	1.72 ± 0.21 ^a^	1.44 ± 0.07 ^b^	2.27 ± 0.34 ^a^	1.78 ± 0.38 ^b^
SGR (% day^−1^)	2.13 ± 0.14 ^a^	2.37 ± 0.05 ^b^	1.78 ± 0.19 ^a^	2.10 ± 0.29 ^b^
Survival (%)	76.67 ± 5.78 ^a^	82.50 ± 2.67 ^b^	50 ± 5.48 ^a^	60 ± 8.94 ^b^

SGR, specific growth rate; FCR, feed conversion ratio. Superscript letters ^a,b^ indicate that group means within a row of the specific experiment without a common superscript differ significantly (*p* < 0.05) based on *t*-test or Kruskal–Wallis test. CON = diet without probiotic supplemented, Pro-3g = diet with probiotic supplementation, i.e., AquaStar^®^ (3 g/kg).

## Data Availability

The data presented in this study are available on request from the corresponding author.
